# Predictors of community acquired childhood pneumonia among 2–59 months old children in the Amhara Region, Ethiopia

**DOI:** 10.1186/s12890-021-01548-w

**Published:** 2021-05-25

**Authors:** Muluken Genetu Chanie, Mequannent Sharew Melaku, Melaku Yalew, Mastewal Arefaynie, Gedamnesh Bitew, Erkihun Tadesse Amsalu, Bereket Kefale, Amare Muche, Zinabu Fentaw, Reta Dewau, Bezawit Adane, Yitayish Damtie, Wolde Melese Ayele, Gojjam Eshetie Ewunetie, Metadel Adane

**Affiliations:** 1 Department of Health Systems and Policy, School of Public Health, College of Medicine and Health Sciences, Wollo University, Dessie, Ethiopia; 2 Department of Health Informatics, Institute of Public Health, College of Medicine and Health Sciences, University of Gondar, Gondar, Ethiopia; 3 Department of Reproductive Health, School of Public Health, College of Medicine and Health Sciences, Wollo University, Dessie, Ethiopia; 4 Department of Epidemiology and Biostatistics, School of Public Health, College of Medicine and Health Sciences, Wollo University, Dessie, Ethiopia; 5 Department of Medical Laboratory Science, Dembya Primary Hospital, Gondar, Ethiopia; 6 Department of Environmental Health Sciences, College of Medicine and Health Sciences, Wollo University, Dessie, Ethiopia

**Keywords:** Amhara region, Case–control, Children, Community acquired pneumonia, Predictors

## Abstract

**Background:**

Worldwide, pneumonia is the third leading cause of death in under 5 years children. Ethiopia is ranked 4th out of 15 countries having the highest burdens of the death rate among under-five children due to pneumonia. Regardless of this fact, efforts to identify determinants of pneumonia have been limited yet in Amhara region. This study was aimed to identify predictors of community-acquired childhood pneumonia among 2–59 months old children in the Amhara region, Ethiopia.

**Methods:**

Facility-based case–control study was conducted in the Amhara region from June 4 to July 15, 2018, among 28 health centers distributed across the region. The total sample size used was 888 (296 cases and 592 controls) children whose age were 2–59 months. At first, multistage sampling technique was employed. Data were collected on a face-to-face interview. Epi data v. 4.6 for data entry and statistical packages for social sciences version 23 for data analysis were used. Multivariable logistic regression analyses were used to test the associations between the study variables at P-value < 0.05 with 95% CI. As a result, determinants were identified for CAP.

**Results:**

Among 888 enrolled children (296 cases and 592 controls), who experienced a community-acquired pneumonia had an increased risk of maternal age of 18–24 years (AOR 0.03, at 95%CI (0.01, 0.14), Government employee (AOR 0.19, at 95% CI (0.07,0.54), lack of separate kitchen (AOR 5.37; at 95% CI (1.65, 17.43), history of diarrhea in the past two weeks (AOR 10.2; at 95% CI (5.13, 20.18), previous respiratory tract infections (AOR 8.3, at 95% CI (3.32, 20.55) and history of parental asthma (AOR 4.9, at 95% CI (2.42, 10.18).

**Conclusion:**

Maternal age of 18–24 years and government employee, lack of separate kitchen, history of diarrhea in the past two weeks; previous respiratory tract infection and history of parental asthma were found statistically significant. Health personnel’s needs to focus on creating awareness to the community on the merit of the separate kitchen for reduction of Community-acquired childhood pneumonia, and focus on prevention and management of childhood diarrheal and acute respiratory tract infections.

## Background

Community-acquired childhood pneumonia (CAP) is an acute infection of the lower respiratory tract that starts outside or is identified within 48 h after admission to the hospital in a child who has not resided in long-term care for 14 days or more before admission [1].

The key symptom of CAP in children are fast breathing. World Health Organization (WHO) has defined fast breathing as a respiratory rate of > 60/min for infants less than 2 months age; > 50/min for infants of 2–12 months age, and > 40/min for children more than 12–59 months age. Pneumonia is caused by several infectious causes, including viruses, bacteria, and fungi. The commonest bacterial cause is Streptococcus pneumonia [1, 2].

Early identification of childhood pneumonia is a crucial technique for the prevention and reversal of the impacts of the condition. Radiological and other laboratory investigations of the etiology are the confirmatory methods to establish the diagnosis of pneumonia, even if these investigations are largely unaffordable in the low socio-economic areas, like Ethiopia. In this areas, the recommended diagnosis approach is, therefore, to rely on the clinical presentation of pneumonia [3, 4].

CAP is the leading cause of morbidity and mortality among under-five children, across the world [5]. The incidence of CAP in under-five children is 0.29 episodes per child-year, which equates to 151.8 million cases annually in developing countries; a further 4 million cases occur in developed countries. Fifteen countries contribute 74% of the world's annual pneumonia cases including Ethiopia [6].

Pneumonia is the leading cause of childhood death in low-income countries where it accounts for up to 21% of deaths in children [8]; and its rate ranges from 60 to 100/1000 live births, 1/5 of these deaths were due to pneumonia [5]. In Sub-Saharan Africa, the estimated proportion of death in children attributed to pneumonia was 17–26% [7]. Approximately 2400 children were expected to die each day and it accounted for approximately 896,000 under-five deaths. An estimated 880,000 under-five children deaths were reported in 2016 globally, where most of the deaths were children < 2 years old [7].

Ethiopia is ranked fourth (84 deaths per 1000) out of the 15 highest clinical pneumonia death rate countries [8]. Similarly, pneumonia is the third prevalent, 20%, acute respiratory tract infection for 2–59 months children in urban settings of the Amhara region [9].

Pneumonia death is strongly associated with poverty-related factors like under-nutrition, lack of safe water and sanitation, indoor air pollution, and inadequate access to health care. Almost half of the pneumonia deaths were associated with air pollution. The impact of indoor air pollution kills more children globally than outdoor air pollution [10, 11]. Outdoor air pollutants (PM10, NO2, O3, CO, and SO2), diurnal temperature range, and tree and weed.

pollen, human rhinovirus, and influenza virus had significant effects on children respiratory tract [23]. Poverty, lower-income, low parental educational level, low birth weight, malnutrition, and lack of breastfeeding are risk factors for acquiring respiratory infectious diseases in developing countries [12].

The rate of all causes of death in under-five children has been reduced by more than half worldwide since 1990, from 91/1000 to 43/1000 in 2015 live births. Although that is an enormous achievement, pneumonia-related under-5 deaths remain stubbornly high. In 2015, this infection was responsible for nearly one of every four deaths that occurred in children under 5 years [13].

Controlling the continuing threat of pneumonia is one of the major health priorities of the FMoH of Ethiopia for which this study aimed to contribute its part. The result was used to ensure the continuum of care so that healthy preschool children transformed into healthy adolescents. Therefore, this study was aimed to identify predictors of community-acquired childhood pneumonia among 2–59 months old children in the Amhara region, Ethiopia, 2018.

## Methods

### Study design and setting

The study was conducted in the Amhara region from June 4 to July 15, 2018, and facility-based case–control study design was employed. This region is located 521 km away from Addis Ababa, which is the capital city of Ethiopia. The total population of the region was estimated to be 32,219,978 in 2017 based on projections from the 2007 population and housing census of which females account for 49.9% of the total population. Out of all 4,349,697 (13.5%) were children under 5-years-old. The Amhara region has 11 zones, 140 woredas, 3429 kebeles (smallest administrative unit), and 96 public hospitals, 826 health centers, and 12,713 health professionals.

### Population

Both cases and controls were all children age 2 months to 5 years in the region.

### Cases

Cases were children of age 2–59 months who were found to have non-complicated (non-admitted) community-acquired pneumonia (CAP) as defined by the World Health Organization (WHO) Integrated Management of Childhood Illness (IMNCI) guideline adopted by the Ethiopian Government since 2001 [11], and it was verified by a physician diagnosis.

### Controls

Controls were children of age 2–59 months who had no pneumonia (clinical pneumonia ruled out by physician) but presented for other services like immunization, growth monitoring, Vitamin A supplementation & de-worming.

### Operational/standard definitions

Under-five children: Children of age 2–59 months, where infants less than 2 months were not included because at the age of < 2 months, the case is not a diagnosis as pneumonia [9].

Community-acquired pneumonia (CAP): acute infection (of less than 14 days duration), acquired in the community, of the lower respiratory tract leading to cough and fast breathing and/or fever.

Household history of acute lower respiratory tract infection (ALRTI): a household with a history of pneumonia or bronchitis in the last fifteen days before data collection.

Household history of acute upper respiratory tract infection (AURTI): a child whose family has a history of ear infection, common cold, tonsillitis, or pharyngitis in the last fifteen days prior to data collection.

### Sample Size determination and Sampling procedure

The Sample size was determined based on double population proportions formula using EPI info (statcal.) version 7 software. It was conducted by taking the following assumptions: 95% confidence level, 80 percent power, and 14.3% controls exposed, and OR of 2.0 [17]. Parental smoking was selected because it was the exposure variable that gave the highest sample size of cases and controls among the other variables from previous studies. Finally, the total sample size used (after adding 2 design effect) was 888 (296 cases and 592 controls at case 1:2 control ratio). At first multistage sampling technique was used. In the region, there were 11 zonal administrations (3 of them were included). In these three zones, there were 89 districts (7 of them were selected by lottery method). In these seven districts, there were 47 health centers where 28 of them were included for this study (by lottery method). Based on the number of 2–59 months old children found who visited each health center in the previous year prior to the data collection period from the under-five outpatient department (OPD) register book, the total sample size was proportionally allocated to the 28 health centers. Screening for pneumonia in under-five OPD and the maternal and child health department were performed based on the Integrated Management of Newborn and Childhood Illness (IMNCI) guideline. Then Controls were selected from Growth monitoring and expanded programs on immunization (EPI) units of the health centers after excluding pneumonia by the assigned clinicians.

The total yearly cases (children with CAP) and other children who visited the health centers for different services (controls) were reported monthly through the health management information system (HMIS) of the health centers. Each health center annual report was summed and divided by 12 to get the average monthly under-five child visit of the health centers.

In each health facility per month multiplied by the total cases (N = 148), divided by the total number of pneumonia cases attended in the entire under-five OPD per month (57 cases). To determine the sample size required from each health center, the average monthly 2–59 months child flow cases and controls were multiplied by the duration of the study period (1 months and a week). Finally, the study subjects were drawn from each selected health facility consecutively. Mothers/Caretakers whod had more than one under-five children were interviewed about the youngest child at the time of the interview.

All cases were considered in all health centers during the study period, and for each case two consecutive controls were used to select controls.

### Data collection tools and procedure

Data collection tools were adopted from different works of literatures reviewed (6, 9, 14–16). Data were collected by IMNCI trained five BSc nurses (unemployed) using a pre-tested, structured, and face-to-face interview. The questionnaires were first prepared in English. The English version questionnaires were translated to the Amharic version and were retranslated to English to check its consistency. Finally, a record review was done to collect information on the height, weight, and zinc supplementation status of children.

### Data quality control

Five data collectors and two experienced supervisors were recruited for the data collection process. The One-day training was given to data collectors and supervisors before the initiation of the data collection process. The questionnaires were pre-tested on 5% (on 30 controls and 15 cases) of the total sample size in Tseda health center which was not included in the main study but has similar socio-demographic characteristics with the study population. The quality of the data were assured by a properly designed and pre-tested questionnaire, proper training of the interviewers and supervisors, proper categorization and coding of the questions. Every day, all questionnaires were reviewed and checked for completeness by the supervisors and principal investigator and the necessary feedback was provided to data collectors the next morning before the actual procedures begin.

### Data management and analysis

During and at the end of data collection, all questionnaires were checked for completeness. Data cleaning was performed, entered into Epi-Data version 4.6, and exported to SPSS version 23 for analysis. The descriptive analyses were presented using frequency tables and percentages. Errors identified at this stage were corrected by reviewing the original data using the code numbers of the questionnaires. Bivariable analysis was conducted to identify individual variables associated with the CAP at 95% CI and P-value ≤ 0.25 [17] were used to analyze the final multivariable logistic regression model. A Backward stepwise regression method was used for selecting the variables. Variables with p-values of ≤ 0.05 were used to identify determinants of CAP. The degree of association between independent and the outcome variables was assessed using an adjusted odds ratio, and their statistical significance was assessed at 95% CI.

### Ethical approval and consent to participate

Ethical clearance was taken from the ethical review committee of Wollo University, College of Medicine and Health Sciences. Letter of permission to conduct the study was obtained from the administrative office of each respective office. Written informed consent was obtained from mother/caregivers prior to data collection. They were informed that participating in the study was voluntary. The right to withdraw from the study at any moment during the interview was assured. No personal identifiers were used on the data collection form. The recorded data was not accessed by a third person except the principal investigator, and was kept confidentially and anonymously. This study did not conduct experiment involving humans. All the procedures of this study were conducted according to the Helsinki declaration of ethical approval and consent to participate.

## Results

### Socio-demographic characteristics of the respondents

Overall, 888 children (296 cases and 592 controls) were enrolled in the study making a 100% response rate for both study groups. The mean (± SD) age of the children was 24.16 (± 14.10) months and 23.94 (± 15.30) months for cases and controls, respectively. The mean (± SD) age of mothers/caregivers was 29.19 (± 4.18) years and 25.77 (± 5.06) years for cases and controls respectively. Of these enrolled children, 210 (70.9%) cases and 470 (79.4%) controls were permanent urban residents. Female children account for 140 (47.3%) of cases and 310 (52.4%) of controls. Concerning to the household monthly income of the respondents, a large proportion of mothers 206(69.59%) in the cases group reported a household monthly income of between 2500 and 6000 ETB; while mothers in the controls group 426 (72.0%) reported that 2500–6000 ETB per month. A number of children greater than 3 years living in the households was 226 (76.4%) of the cases and 414 (69.9%) of controls were lived in the households (Table 1).

**Table 1:** Socio-demographic characteristics of children’s 2–59 months age in Amhara region, Northwest Ethiopia, 2018 (n = 296 case and 592 controls)

Variables	Pneumonia status	
	Cases, n (%)	Controls, n (%)
Residence		
Urban	210 (70.9)	470 (79.4)
Rural	86 (29.1)	122 (20.6)
Sex of child		
Male	156 (52.7)	282 (47.6)
Female	140 (47.3)	310 (52.4)
Age of the child, months		
2–11	62 (20.9)	162 (27.4)
12–23	92 (31.1)	170 (28.7)
24–35	64 (21.7)	102 (17.2)
36–47	48 (16).2	78 (13.2)
48–59	30 (10.1)	80 (13.5)
Age of the mother, years		
18–24	38 (12.8)	266 (44.9)
25–34	234 (79.1)	298 (50.4)
≥ 35	24 (8.1)	28 (4.7)
Educational status of mother		
Primary 1–4	64 (21.6)	116 (19.6)
Primary 5–8	110 (37.1)	114 (19.2)
Secondary 9–12	94 (31.8)	216 (36.5)
College/higher education	28 (9.5)	146 (24.7)
Marital status of mother		
Married	260 (87.8)	566 (95.6)
Divorced/widowed	36 (12.2)	26 (4.4)
Educational status of father		
Primary 1–4	52 (16.9)	104 (15.5)
Primary 5–8	108 (36.5)	158 (26.7)
Secondary 9–12	72 (24.3)	104 (17.6)
College/higher education	64 (21.6)	226 (38.2)
Mother occupation		
Housewife	192 (64.9)	296 (50.0)
Student	32 (10.8)	76 (12.8)
Government employee	36 (12.2)	152 (25.7)
Merchant	24 (8.1)	52 (8.8)
Others	12 (4.0)	16 (2.7)
House hold monthly income		
< 2500 Birr	32 (10.82)	84 (14.1)
2500–6000Birr	206 (69.59)	426 (72.0)
> 6000Birr	58 (19.59)	82 (13.9)
Number of under-five children in the family		
1–2 children	70 (23.6)	178 (30.1)
> 3 children	226 (76.4)	414 (69.9)

Others = refers to private employee, NGO employee and daily laborer

### Characteristics of the respondents

Regarding the housing status of respondents, 280 (98.6%) had a roof with corrugated iron sheets for both study groups. More than half of the cases 176 (59.5%) and controls 298(50.3%) had an earthen floor and their wall was made of wood with mud. One hundred and seventy-two (58.1%) cases and 106 (17.9%) of controls came from less than 5 km to health centers. Two hundred and eighty-six (96.6%) of cases and 578 (99.3%) of controls houses had windows. The main types of fuel used for cooking were wood and dung (in 50.7% of the households of cases). About 146 (49.3%) of cases and 338 (57.1%) of controls were carried their child on back during cooking. About 230 (77.7%) of cases and 420 (70.9%) of controls were cared for by their parents. Regarding sleeping rooms, less than three persons in the family share bed during sleeping for cases were 272 (91.9%) and controls 570 (96.3%) (Table 2).

**Table 2:** Home based characteristics of children 2–59 months age in Amhara region, Northwest Ethiopia, 2018 (n = 296 case and 592 controls)

Variables	Pneumonia status	
	Cases, n (%)	Controls, n (%)
Main material of roof		
Thatched roof	16 (1.4)	8 (1.4)
Corrugated iron	280(98.6)	584 (98.6)
Main material of floor		
Earth with mud/others	176 (59.5)	298 (50.3)
Cement/brick	120 (40.5)	294 (49.7)
Distance from Health Centers		
< 5 km	172(58.1)	106 (17.9)
5–10 km	100 (33.8)	348 (58.8)
> 11 km	24 (8.1)	138 (23.3)
Presence of Separate kitchen in the house		
Yes	252 (85.1)	576 (97.3)
No	44 (14.9)	16 (2.7)
N o and Open of kitchen windows during cooking		
Yes	188 (63.5)	572 (96.6)
No	108 (36.5)	20 (3.4)
Separate Cattle room		
Yes	72 (24.3)	78 (13.2)
No	224 (75.7)	514 (86.8)
Presence of window in the house		
Yes	286 (96.6)	578 (99.3)
No	10 (3.4)	14 (0.7)
Source of water		
Pipe water	216 (73.0)	484 (83.4)
Protected spring water	80 (27.0)	98 (16.6)
Cooking practices in the houses		
Wood/dung	150 (50.7)	332 (56.1)
Charcoal	20 (6.8)	34 (6.7)
Stove/gasoil	22 (6.8)	26 (3..4)
Electricity	104 (35.7)	200 (33.8)
Mother/caregiver carrying child at the back while cooking		
Yes	146 (49.3)	338 (57.1)
No	150 (50.7)	254 (42.9)
Child caring practice in the house		
Parental care	230 (77.7)	420 (70.9)
Housemaid	66 (22.3)	172 (29.1)
Persons share bed during sleeping		
Three or less	272 (91.9)	570 (96.3)
More than Three	24 (8.1)	22 (3.7)
UHEP HH Status		
Trained and graduated	8 (2.1)	36 (6.1)
On training	144 (49.3)	318 (53.7)
Not Packaged	144 (48.6)	238 (40.2)

### Nutritional characteristics of the respondents

Regarding zinc supplementation during diarrhea, mothers reported that their children were not ever supplemented with zinc for 230 (80.4%) of cases and 448(92.6%) of controls groups. Two hundred and sixty-four (90.5%) of cases and 556 (93.9%) of controls started complementary feeding after 6 months of birth. Regarding nutritional status 286 (96.6%) of cases and 570 (96.3%), controls were not stunted but 12 (4%) of cases and 21 (3.5%) of controls were wasted (Table 3).

**Table 3:** Nutritional status characteristics of children 2–59 months age in Amhara region, Northwest Ethiopia, 2018 (n = 296 case and 592 controls)

Variables	Pneumonia status	
	Cases, n (%)	Controls, n (%)
Zinc Supplementation		
Yes	58 (19.6)	44 (7.4)
No	238 (80.4)	448 (92.6)
Current BF Status		
Yes	168 (56.8)	320 (54.1)
No	128 (43.2)	272 (45.9)
Birth to 6 months of breast feeding		
Exclusive BF	280 (97.9)	580 (99.3)
Non-Exclusive BF	16 (2.1)	12 (0.7)
Duration of BF		
No BF	8 (2.7)	14 (2.4)
12 months or less	142 (47.9)	178 (30.1)
13–24 months	126 (42.6)	350 (59.1)
> 24 months	20 (6.8)	50 (8.4)
Begin Complementary feeding		
Before 6 months	28 (9.5)	36 (6.1)
After 6 months	264 (90.5)	556 (93.9)
Nutritional status of the child		
Height for Age (HFA)		
Not stunting	286 (96.6)	570 (96.3)
Stunting	10 (3.4)	22 (3.7)
Weight for Age (WFA)		
Normal	282 (95.3)	566 (95.6)
Underweight	14 (4.7)	26 (4.4)
Weight for Height (WFH)		
Not Wasting	284 (96.0)	571(96.5)
Wasting	12 (4.0)	21 (3.5)
MUAC		
< 11 CM	98 (33.1)	160 (27.0)
11–11.9 CM	96 (32.4)	194 (32.8)
12 CM and above	102 (34.5)	238 (40.2)

### Childhood illnesses and related care practices associated with community acquired-pneumonia

About 72 (24.3%) of cases and 141 (23.8%) of controls had currently diarrheal illnesses. Regarding the vaccination statuses of children, the majority of cases 232 (78.4%) and controls 448 (75.7%) received a full dose of pentavalent vaccine. Two hundred and forty-four (82.4%) cases and 482 (81.4%) controls received the measles vaccine. Two hundred and eighty-one (95.0%) of cases and 554 (93.6%) controls received a full dose of the pneumococcal conjugate vaccine (Table 4).

**Table 4:** Common Childhood illnesses & related care practices of children 2–59 months age in Amhara region, Northwest Ethiopia, 2018 (n = 296 case and 592 controls)

Variables	Pneumonia status	
	Cases, n (%)	Controls n (%)
Diarrhea in the Last 2 weeks		
Yes	117 (39.5)	74 (12.5)
No	179 (60.5)	518 (87.5)
Current Diarrhea illness		
Yes	72 (24.3)	141 (23.8)
No	224 (75.7)	451 (76.2)
URTI in the last 2 weeks		
Yes	56 (18.9)	150 (25.3)
No	240 (81.1)	442 (74.7)
History of measles illness		
Yes	8 (2.7)	4 (0.67)
No	288 (97.3)	588 (99.33)
History of Lower Respiratory Infection in the last 2 weeks		
Yes	62 (20.9)	24 (4.1)
No	234 (79.1)	568 (95.9)
History of Parental Asthma in the family		
Yes	75 (23.9)	51 (8.6)
No	221 (76.1)	541 (91.4)
A child Received Pentavalent Vaccine		
No vaccinated	8 (2.7)	7 (1.2)
1–2 dose taken	56 (18.9)	137 (23.1)
Full dose taken	232 (78.4)	448 (75.7)
A child Received Measles Vaccine		
Yes	244 (82.4)	482 (81.4)
No	52 (17.6)	110 (18.6)
A child Received PCV Vaccine		
Yes	281 (95.0)	554 (93.6)
No	15 (5.0)	38 (6.4)

### Predictors of community-acquired pneumonia

In the bi-variable logistic regression analysis, 13 variables were eligible at p-value <  = 0.25 and fitted to the multivariable logistics regression model. In the multivariable logistic regression model, six determinant factors of CAP in children between 2 months and 5 years were identified. The finding revealed that a child born from a mother whose age was between 18 and 24 years had a 97% less chance to develop CAP as compared to a child born to a mother whose age was 35 years or above (AOR 0.03, 95% CI 0.01, 0.14). Children who were born from government employees had a 98% less chance to develop childhood community-acquired pneumonia as compared to children whose mothers were worked with housewife occupation (AOR 0.19, 95% CI 0.07, 0.54). This result showed that children from those household who has no separate kitchen for cooking purposes were 5.4 times increased risk of childhood community-acquired pneumonia as compared to children from those households who had separate kitchen from the main house (AOR 5.4; 95%CI 1.65, 17.43).

Children who had a history of diarrhea in the past fifteen days were 10.2 times more likely to develop CAP compared to their counterparts (AOR 10.2; 95%CI 5.13, 20.18). Children from households with a history of ALRI within the past fifteen days prior to data collection were 8.3 times more likely to develop CAP compared to their counterparts (AOR = 8.3, 95%CI: 3.32, 20.60). Likewise, Children from households with a history of asthma were 4.9 times more likely to develop CAP than children whose parents had no history of asthma (AOR 4.9, 95% CI 2.42, 10.18) (Table 5).

**Table 5:** Determinant factors of Community Acquired childhood Pneumonia among children age 2–59 months in Amhara region, Ethiopia, 2018 (n = 296 case and 592 controls)

Variables	Pneumonia status		OR at 95% CI	
	Cases, n (%)	Controls, n (%)	COR (95%CI)	AOR (95%CI)
Age of the mother (years)				
18–24	38 (12.8)	266 (44.9)	0.17 (0.07, 0.41)*	0.03 (0.01, 0.14)*
25–34	234 (79.1)	298 (50.4)	0.92 (0.41, 2.06)	0.51 (0.16, 1.62)
>= 35	24 (8.1)	28 (4.7)	1.0	1.0
Maternal occupation				
Housewife	192 (64.9)	296 (50.0)	1.0	1.0
Student	32 (10.8)	76 (12.8)	0.65 (0.34, 1.23)	2.03 (0.73, 5.64)
Gov’t employee	36 (12.2)	152 (25.7)	0.37 (0.21, 0.65)*	0.19 (0.07, 0.54)*
Merchant	24 (8.1)	52 (8.8)	0.71 (0.34, 1.48)	0.96 (0.37, 2.52)
Others	12 (4.0)	16 (2.7)	1.16 (0.39, 3.44)	2.15 (0.47, 9.68)
Separate kitchen with available windows during cooking				
No	108 (36.5)	20 (3.4)	16.4 (8.05, 33.55)**	5.37 (1.65, 17.43)**
Yes	188 (63.5)	572 (96.6)	1.0	1.0
Diarrhea in the Last 2 weeks				
Yes	117 (39.5%)	74 (12.5%)	4.02 (2.49, 6.50)**	10.2 (5.13, 20.18)**
No	179 (60.5%)	259 (87.5%)	1.0	1.0
History of Lower Respiratory Infection in the last 2 weeks				
Yes	62 (20.9%)	24 (4.1%)	6.27 (3.11, 12.6)**	8.3 (3.32, 20.6)**
No	234 (79.1%)	568 (95.9%)	1.0	1.0
History of Parental Asthma in the family				
Yes	75 (25.3%)	51 (8.6%)	3.59 (2.08, 6.19)**	4.9 (2.42, 10.18)**
No	221 (74.7%)	541 (91.4%)	1.0	1.0

## Discussion

Pneumonia in under-five children is a leading cause of morbidity and mortality in developing countries including Ethiopia. Knowledge of the possible determinant factors is important for proper management and prevention strategy of Community-Acquired Pneumonia. Among the socio-demographic factors, the key predictors for pneumonia among under-five children in this study were child’s maternal age between 18 and 24 years were protective factor against CAP. A similar case–control study that was conducted in Kersa District, Southwest Ethiopia reported maternal age < 18 years was more likely to develop CAP [14]. A study in Thailand, Brazil, and Southeast Asian countries reported that young maternal age was a risk factor for developing childhood pneumonia [11, 15] that contradicts the current study result. The possible reason for this variation might be due to differences in maternal age labeling that different studies used, differences in screening criteria for pneumonia among under 2 months up to 5 years old children, differences in population and geography, and differences in sample size. In this study, the sample size was high as compared with the above studies. The screening criteria used for pneumonia in this study was the Integrated Management of Newborn and child care guideline, but pneumonia was diagnosed by using clinical presentation and imaging machines in Bangladesh, Southern Brazil, and Brasilia [14, 16, 17]. The other explanation might be due to better practice of the younger mothers on their child care, and a number of individuals involved in child-caring practices at home. Additionally, in mothers whose age between 18 and 24 years, they have an opportunity to care their children with their peer groups than older mothers, which could lead to a preventive action for occurrence of the community-acquired pneumonia.

This study was also found child’s mothers who were governmental employees were protective against CAP. This result was in line with the study findings in India, Baghdad/Iraq [11, 13]. Possible reasons might be mothers spend less or no time cooking food for their family while carrying their children on their back, which reduces exposure to indoor air pollution, and mothers working in professional or technical occupations are likely to have literacy or awareness which may also directly or indirectly contribute to preventing their children from community-acquired pneumonia.

Households with no separate kitchens were five times more likely to acquire CAP as compared to their counterparts. This study was consistent with the studies conducted in Este town, Northwest Ethiopia [18], Nigeria [16], Ndola (Zambia) [19], and Kenya [20]. Therefore, children whose households cooking rooms is within the main house where the child sleep were found to have a threefold greater risk of ARIs than children belonging to a household with separate kitchen [13]. In this case risk of indoor air pollution is high which increases the vulnerability of children to acquire ARI including pneumonia.

Children who had a history of diarrhea in the past fifteen days were ten times more likely to develop community-acquired pneumonia compared to their counterparts. Similar studies conducted in Urban Areas of Oromia special Zone of Amhara Region, Tigray Ethiopia and Zimbabwe [10, 20, 21] reported different findings with current findings, the difference might be due to variation in methods. This can be explained by the fact that children who have a concomitant illness like diarrhea may have a lowered immunity, making them more susceptible to diseases like pneumonia.

According to this study children who had a past history of ALRIs in any household member in the past two weeks prior to the investigation were eight times higher risk of having CAP. It is consistent with an institutional-based study conducted at Kemise, Oromia zone, Amhara Region which showed that children from households with a history of ALRI within the past fifteen days prior to data collection were three times more likely to develop pneumonia compared to their counterparts [10]. The possible explanation for lower respiratory tract infections were contagious and are easily transmittable from household contacts to children. These infections were often viral in origin and which may be seen as the consequence of progression from milder forms of lower respiratory tract to predispose children to pneumonia, severity of the disease also depends on virulence and load of the pathogen; the load is usually higher when the infection is from a household contact [22].

Finally, a positive family history of asthma or allergy had a four times higher risk of developing CAP as compared to families with no history of asthma. This result coincides with the study result in India [20], Baghdad/Iraq [13]. The reason for the difference might be due to differences in sampling, study setting, and seasonal variations in these two study areas. another similar study was reported that maternal history of asthma increases the risk of severe LRTIs in the first year of life [8]. Respiratory tract infections are easily transmitted from household contacts to children might be a reason. The severity of the disease depends on the virulence and load of the pathogen; where the agent load is usually higher when the infection is from a household contact [7]. The implication for the above explanatory factors were noted that mothers occupation, lack of separate kitchen, and other illness in the family had a significant association with determinants of childhood CAP. It was also shown that employed mothers /caretakers had adequate knowledge when compared to others. This may be due to employed mothers/caretakers had communication access with many people including their colleagues with different experiences.

### Limitation of the study

Diagnosis of pneumonia was based on clinical WHO IMNCI classification guidelines, which could introduce misclassification bias. Another limitation may be an institution-based case–control study that can limit the generalizability.

## Conclusions

The result of this study identified determinants of community-acquired pneumonia among 2–59 months of children. Age of mothers, mother’s occupational status, and separate kitchen, history of diarrhea, ALRI, and asthma were found to be significant factors for CAP. All public health centers should promote early prevention and treatments of Diarrhea, ARTI of children in the health facility and at household level. They need to create awareness about the power of separate kitchens in the reduction of CAP in children.

## Data Availability

All the data supporting the findings are within the manuscript. Additional detailed information and dataset are available from the corresponding author on reasonable request.

## Abbreviations

ALRTI: Acute lower respiratory tract infection
AOR: Adjusted odds ratio
AURTI: Acute upper respiratory tract infection
BSc: Bachelor of Science
CAP: Community acquired pneumonia
CI: Confidence Interval
EPI: Expanded program on Immunization
Epi-data: Epidemiological data
Epi-info: Epidemiological information
ETB: Ethiopian Birr
FMoH: Federal Ministry of Health
HMIS: Health management information system
IMNCI: Integrated management of neonatal and child illnesses
OPD: Outpatient department
OR: Odds ratio
SD: Standard deviation
SPSS: Statistical packages for social sciences
WHO: World Health Organization

## References

[1] Carol M, Glenn PM. Concepts of altered health states. New York: Walters Kluwer Health Lippincott Williams & Wilkins, 676–80.

[2] WHO. Revised WHO classification and treatment of childhood pneumonia at health facilities. 2014. 25535631

[3] World Pneumonia Day 2016. Available from: http://www.who.int/mediacentre/factsheets/fs331/en/

[4] Markos Y, Dadi AF, Demisse AG, Habitu YA, Derseh BT, Debalkie G (2019). Determinants of under-five pneumonia at Gondar University Hospital, Northwest Ethiopia. J Environ Public Health.

[5] Geleta D, Tessema F, Ewnetu H (2016). Determinants of community acquired pneumonia among children in Kersa District, Southwest Ethiopia. J Pediatr Neonatal Care.

[6] Chen J, Hu P, Zhou T, Zheng T, Zhou L, Jiang C (2018). Epidemiology and clinical characteristics of acute respiratory tract infections among hospitalized infants and young children in Chengdu, West China. BMC Pediatr.

[7] Getaneh S, Alem G, Meseret M, Miskir Y, Tewabe T, Molla G (2019). Determinants of pneumonia among 2–59 months old children at Debre Markos referral hospital, Northwest Ethiopia. BMC Pulm Med.

[8] Hassena S, Getachewa M, Eneyewa B, Keleba A, Ademasa A, Berihuna G (2020). Determinants of acute respiratory infection (ARI) among under-five children in rural areas of Legambo District, South Wollo Zone, Ethiopia. Int J Infect Dis.

[9] Abel Fekadu D, Yigzaw K, Zelalem B. Determinants of pneumonia in children aged two months to five years in Urban Areas of Oromia Zone, Amhara Region, Ethiopia. Open Access Li;2014.

[10] Alemayehu S, Kidanu K, Kahsay T, Kassa M (2019). Risk factors of acute respiratory infections among under five children attending public hospitals in southern Tigray, Ethiopia. BMC Pediatr.

[11] Razanajatovo NH, Guillebaud J, Harimanana A, Rajatonirina S, Ratsima EH, Andrianirina ZZ (2018). Epidemiology of severe acute respiratory infections from hospital-based surveillance in Madagascar. PLoS ONE.

[12] Mangen M-JJ, Huijts SM, Bonten MJM, Wit GAD (2017). The impact of community-acquired pneumonia on the health-related quality of-life in elderly. BMC Infect Dis.

[13] UNICEF (2016). One is too many: ending child deaths from pneumonia and diarrhea.

[14] Geleta D, Tessema F, Ewnetu H (2016). Determinants of community acquired pneumonia among children in Kersa District, Southwest Ethiopia: facility based case control study. J Pediatr Neonatal Care..

[15] Federal Democratic Republic of Ethiopia (FDRE). EDHS 2016 Final report. (Addis Ababa, Ethiopia).

[16] Fekadu GA, Terefe MW, Alemie GA (2014). Prevalence of pneumonia among under- five children in Este Town and the surrounding Rural Kebeles, Northwest Ethiopia: a community based cross sectional study. Sci J Public Health..

[17] Bendel RB, Afifi AA (1977). Comparison of stoping rules in forward “stepwise regression. J Am Stat Assoc.

[18] Childhood illnesses. Fonseca Limaet al BMC Pediatrics [Internet]. 2016;16(15). Available from: www.biomedcentral.com/submit

[19] UNICEF, Estimates of child cause of death, acute respiratory infection [Internet]. 2015. Available from: https://data.unicef.org/child-health/pneumonia.html

[20] UNO (2015). Sustainable Development Goals (SDG). [Internet]. Available from: http://www.un.org/sustainabledevelopment/health/

[21] World Health Organization and UNICEF. Fulfilling the health agenda for the women and children. 2015. (WHO, 2014).

[22] Fakunle AG, Ogundare JO, Adelekan AL, Bello TA (2017). Household cooking practices as risk factor for acute respiratory infections among hospitalized under-5 children in Ibadan, Nigeria. IOSR J Environ Sci Toxicol Food Technol.

[23] Lee SW, Yon DK, James CC, Lee S (2019). Short-term effects of multiple outdoor environmental factors on risk of asthma exacerbations: age-stratified time-series analysis. J Allergy Clin Immunol..

